# Effects of High-Dose Cyclophosphamide on Ultrastructural Changes and Gene Expression Profiles in the Cardiomyocytes of *C57BL/6J* Mice

**DOI:** 10.3390/diseases12050085

**Published:** 2024-04-27

**Authors:** Takuro Nishikawa, Emiko Miyahara, Ieharu Yamazaki, Kazuro Ikawa, Shunsuke Nakagawa, Yuichi Kodama, Yoshifumi Kawano, Yasuhiro Okamoto

**Affiliations:** 1 Department of Pediatrics, Graduate School of Medical and Dental Sciences, Kagoshima University, Kagoshima 890-8520, Japan; k5424258@kadai.jp (E.M.); ngawa@m2.kufm.kagoshima-u.ac.jp (S.N.); yuichik18@gmail.com (Y.K.); y-kawano@kirishima-mc.jp (Y.K.); okamoto@m2.kufm.kagoshima-u.ac.jp (Y.O.); 2 BML Inc. Research Institute, Kawagoe 350-1101, Japan; mame.ieharu@nifty.com; 3 Department of Clinical Pharmacotherapy, Hiroshima University, Hiroshima 734-8553, Japan; ikawak@hiroshima-u.ac.jp

**Keywords:** cardiotoxicity, cyclophosphamide, functionally important gene, gene expression profiling, ultrastructural aberration

## Abstract

The pathogenesis of cyclophosphamide (CY)-induced cardiotoxicity remains unknown, and methods for its prevention have not been established. To elucidate the acute structural changes that take place in myocardial cells and the pathways leading to myocardial damage under high-dose CY treatments, we performed detailed pathological analyses of myocardial tissue obtained from *C57BL/6J* mice subjected to a high-dose CY treatment. Additionally, we analysed the genome-wide cardiomyocyte expression profiles of mice subjected to the high-dose CY treatment. Treatment with CY (400 mg/kg/day intraperitoneally for two days) caused marked ultrastructural aberrations, as observed using electron microscopy, although these aberrations could not be observed using optical microscopy. The expansion of the transverse tubule and sarcoplasmic reticulum, turbulence in myocardial fibre travel, and a low contractile protein density were observed in cardiomyocytes. The high-dose CY treatment altered the cardiomyocyte expression of 1210 genes (with 675 genes upregulated and 535 genes downregulated) associated with cell–cell junctions, inflammatory responses, cardiomyopathy, and cardiac muscle function, as determined using microarray analysis (|Z-score| > 2.0). The expression of functionally important genes related to myocardial contraction and the regulation of calcium ion levels was validated using real-time polymerase chain reaction analysis. The results of the gene expression profiling, functional annotation clustering, and Kyoto Encyclopedia of Genes and Genomes pathway functional-classification analysis suggest that CY-induced cardiotoxicity is associated with the disruption of the Ca^2+^ signalling pathway.

## 1. Introduction

Cyclophosphamide (CY) is an old alkylating agent; it is one of the most frequently used antitumour drugs [1,2]. CY is used not only as an antitumour agent but also as an immunosuppressive agent for the treatment of nephrotic syndrome [3]. Although a variety of conditioning agents are available for hematopoietic stem cell transplantation (HSCT) to treat haematological malignancies, bone marrow failure, or immunodeficiency, most standard treatment regimens and most commonly employed conditioning modalities include a high dose of CY [2]. In recent years, post-transplant high-dose CY therapy has become popular worldwide as a potent graft-versus-host disease prophylaxis, especially in human leukocyte antigen haploidentical HSCT settings [4,5]. Thus, the use of high-dose CY therapy is increasing.

Cyclophosphamide has side effects such as emetogenicity, haematotoxicity, cardiac toxic effects, pulmonary toxicity, liver damage, renal damage, haemorrhagic cystitis, carcinogenicity, and gonadal toxic effects [2]. Cardiotoxicity is a dose-limiting toxicity of CY. It is well documented that acute cardiac failure occurs within a week in a small number of patients receiving high doses of CY, with unpredictable and fatal consequences [5,6,7]. In addition, cyclophosphamide has recently been reported to influence thrombosis [8].

However, the detailed mechanism of the cardiotoxicity of CY is unclear, and preventive measures have not yet been established. Due to the improving outcomes of HSCT, these potentially fatal complications have attracted research interest.

**Figure 1:**
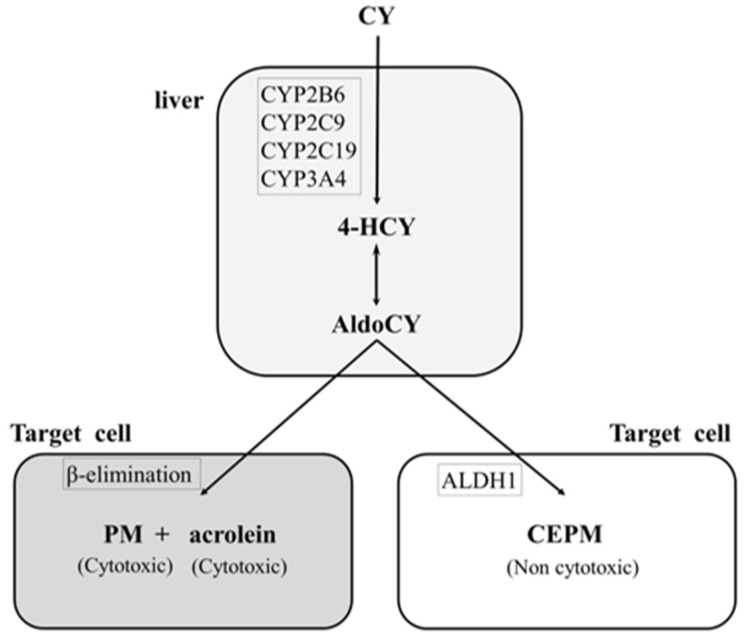
Metabolic pathway of cyclophosphamide. The hepatic cytochrome P-450 enzyme (CYP) system (CYP2B6, 2C9, 2C19, and 3A4) metabolises cyclophosphamide (CY) to 4-hydroxy-cyclophosphamide (4-HCY). Subsequently, 4-HCY is oxidised to its tautomer aldocyclophosphamide (AldoCY), absorbed by cells, and converted to phosphoramide mustard (PM) and acrolein via β-elimination. AldoCY can be oxidised to o-carboxyethylphosphoramide mustard (CEPM) by aldehyde dehydrogenase 1 (ALDH1).

Cyclophosphamide, a prodrug, is converted to the cytotoxic metabolites 4-hydroxycyclophosphamide (HCY) and aldocyclophosphamide (AldoCY) via the cytochrome P-450 (CYP) enzyme system (CYP2B6, 2C9, 2C19, and 3A4) in the liver. Both metabolites then circulate in the body and passively enter other cells. AldoCY undergoes an intracellular β-elimination reaction to form phosphoramide mustard (PM), which exhibits alkylating activity. Acrolein is formed as a by-product of this process. On the other hand, if intracellular aldehyde dehydrogenase 1 activity is high, AldoCY is detoxified to o-carboxyethyl-phosphoramide mustard (CEPM), an inactive metabolite [9,10] (Figure 1).

In our previous study, we evaluated the myocardial cell injury associated with CY by exposing H9c2 cells, a rat cardiac myocardial cell line, to CY metabolites [11,12]. We hypothesised that the exposure would increase myocardial cell injury because of an increase in acrolein levels and apoptosis. Co-exposure to the antioxidant and acrolein scavenger N-acetylcysteine (NAC) [13] suppresses myocardial cell injury induced by the CY metabolites. These results suggest that the protective activity of NAC is due to the inhibition of apoptosis, decreased acrolein production, and increased CEPM production [11]. Subsequently, other investigators have reported that acrolein may mediate CY-induced myocardial damage [14,15,16,17].

Myocardial damage associated with the administration of CY in vivo has not been evaluated using microarray analyses (i.e., elucidating changes in the gene expression profiles). We determined the CY dosage based on the pharmacokinetics of CY and CY metabolites and the clinical course in mice. We then performed a histological examination, including the electron microscopy of the heart. These research processes were first attempts. In this study, we aimed to examine the gene expression profiles of mice treated with CY to elucidate the in vivo mechanism of high-dose CY toxicity using electron microscopy and a comprehensive evaluation of gene expression. As acrolein is expected to be quickly absorbed by proteins and not reach the myocardium when administered externally, this study was primarily conducted by administering CY [18,19].

## 2. Materials and Methods

### 2.1. Reagents

CY (CAS # 6055-19-2) and NAC (Sigma-Aldrich, St. Louis, MO, USA) were dissolved in normal saline (NS). Acrolein (10 mg/mL; AccuStandard^®^, New Heaven, CT, USA) was dissolved in water.

### 2.2. Animals

Five-week-old female *C57BL/6J* mice (Charles River Japan, Kanagawa, Japan) were maintained at 22 ± 2 °C under a 12-/12-h light/dark cycle (light on from 7:00 to 19:00 h); water and food were provided ad libitum. The mice were sedated and anaesthetised. The hypnotic sedative agent medetomidine hydrate (0.7 mg/kg) or analgesic agents midazolam (4 mg/kg) and butorphanol tartrate (5 mg/kg) were mixed and dissolved in saline solution. All experiments were conducted in accordance with the ARRIVE guidelines and the Guidelines for the Proper Conduct of Animal Experiments established by the Science Council of Japan. The animal study was approved by the Institutional Animal Care and Use Committee of Kagoshima University, Kagoshima, Japan. 

### 2.3. Plasma CY, HCY, and CEPM Concentrations after the Administration of High-Dose CY 

To clarify the metabolic kinetics of CY, tail-vein blood samples were collected in EDTA2NA at 1 and 3 h (or 4 h from mice administered 400 mg/kg CY) after a single intraperitoneal administration of CY (400–700 mg/kg); the samples were centrifuged at 700 *g* for 15 min to obtain plasma (Figure S1). The concentrations of CY, HCY, and CEPM were determined using liquid chromatography/tandem mass spectrometry (LC/MS/MS), as previously described [11]. The area under the concentration–time curve (AUC) from zero to infinity was estimated using a non-compartmental analysis with the trapezoidal method in the MOMENT program, based on the available data points.

### 2.4. Histological Examinations 

**Figure 2:**
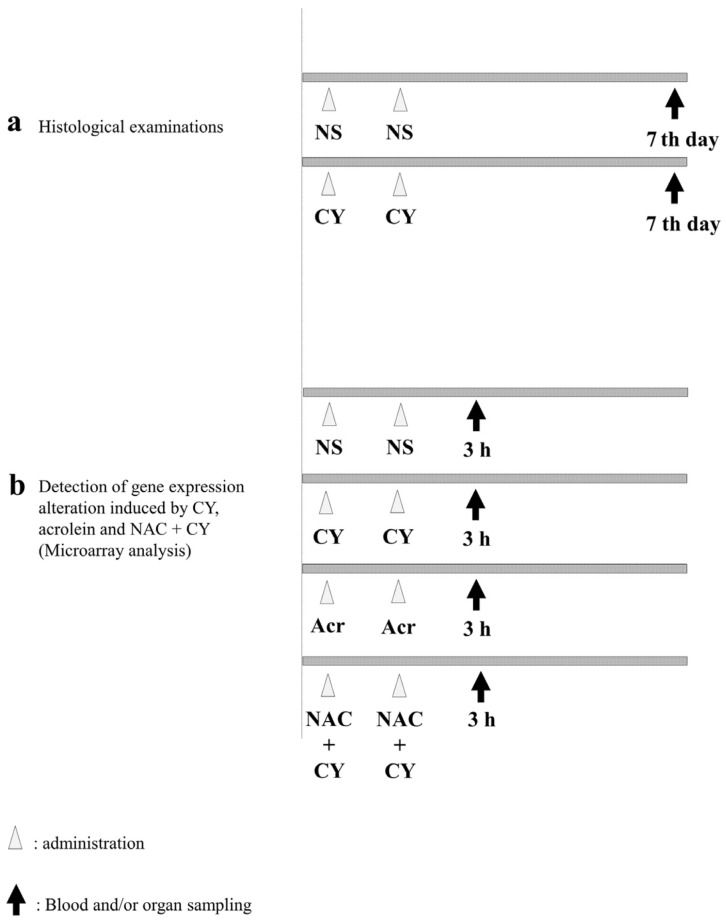
Experimental procedures. (**a**) *C57BL/6J* mice (females aged six weeks) were intraperitoneally treated with normal saline (control) or 400 mg/kg cyclophosphamide (CY) once daily for two consecutive days. Seven days after the last dose, the mice were euthanised under anaesthesia and their hearts were collected. (**b**) *C57BL/6J* mice (females aged six weeks) were intraperitoneally treated with normal saline (control), 400 mg/kg CY, 5 mg/kg acrolein, or 400 mg/kg CY with 200 mg/kg N-acetylcysteine (NAC) once daily for two consecutive days. NAC was administered 2 h before CY administration. Three hours after the last dose, the mice were euthanised under anaesthesia and their hearts were collected.

To examine pathological alterations associated with CY administration, the mice were randomly assigned to CY-treated and control groups. For the mice in the control group, 250 μL of NS was administered intraperitoneally (i.p.) once a day for 2 days. Meanwhile, the mice in the CY-treated group were administered 250 μL of 400 mg/kg CY solution i.p. once a day for 2 days (Figure 2a). The mice in both groups were euthanised under anaesthesia 7 days after the last dose and their hearts were collected. A section of the heart tissue was placed in 4% paraformaldehyde phosphate buffer solution (FUJIFILM Wako Chemicals, Tokyo, Japan) for haematoxylin–Eosin (HE) staining. For the electron microscopy analysis, another section of the heart was placed in 2.5% glutaraldehyde in 0.1 M phosphate buffer (pH 7.4), dehydrated in ethanol, and embedded in Epon 812 (TAAB Laboratories Equipment, Reading, UK). Ultrathin sections (80–90 nm) were obtained, stained with uranyl acetate and lead citrate, and examined using electron microscopy (H-7600; Hitachi, Tokyo, Japan) at an acceleration voltage of 100 kV.

### 2.5. Detection of Gene Expression Alterations 

To elucidate the gene expression alterations induced by CY, the mice were divided into four groups (Figure 2b). NS was used as a negative control. Mice in the CY-treated group were administered 250 μL of 400 mg/kg CY solution i.p. once a day for 2 days. Mice in the acrolein-treated group were administered 5 mg/kg acrolein aqueous solution i.p. Furthermore, to clarify the effect of NAC, 200 mg/kg NAC solution was administered i.p. to mice in the NAC + CY group at 2 h before CY administration (Figure 2b). Three hours after the last dose, all mice were euthanised and their hearts were harvested under anaesthesia. A section of the heart tissue was frozen in liquid nitrogen and stored at −80 °C. 

### 2.6. Total RNA Isolation

The total RNA from the frozen heart tissue was isolated using the NucleoSpin^®^ RNA/Protein kit (MACHEREY-NAGEL, Düren, Germany), quantified using SpectraMax ABS Plus (Molecular Devices, LLC., San Jose, CA, USA), and assessed using an Experion automated electrophoresis station (Bio-Rad Laboratories Inc., Hercules, CA, USA).

### 2.7. Microarray Analysis

The microarray analysis was performed by Cell Innovator Inc. (Fukuoka, Japan). cDNA was amplified and labelled using Low-Input Quick-Amp Labelling (Agilent Technologies, Santa Clara, CA, USA), hybridised using a SurePrint G3 Mouse Gene Expression Microarray 8 × 60 K v2 (Agilent), and scanned using an Agilent scanner. We used Agilent Feature Extraction Software (v9.5.1.1; Agilent Technologies, Santa Clara, CA, USA) to calculate the relative hybridisation intensities and background values.

### 2.8. Data Analysis and Filter Criteria

We normalised the raw signal intensities of the samples (control vs. CY, control vs. acrolein, and control vs. NAC + CY) using the quantile algorithm in the preprocessCore library package [20] in the Bioconductor application [21], selecting probes with P flags in ≥1 sample. We used the normalised signal intensities of each probe to calculate intensity-based Z-scores [22] and ratios (non-log-scaled fold-change) to identify up- and downregulated genes via comparisons between the control (no CY exposure) and experimental samples (CY, acrolein, and NAC + CY). The criteria were a Z-score of ≥2.0 and a ratio of ≥1.5-fold for upregulated genes, and a Z-score of ≤–2.0 and a ratio of ≤0.66 for downregulated genes. Significant over-representations of Gene Ontology (GO) categories and significant pathway enrichment were determined using the Database for Annotation, Visualization, and Integrated Discovery (DAVID, 2021 update) [23], and functional annotation clustering and Kyoto Encyclopedia of Genes and Genomes (KEGG) pathway functional classification analysis were performed [24]. We used MeV software 4.9.0 [25] to generate a heatmap, and genes were sorted using the hierarchical clustering method, with colours used to indicate the distance from the median of each row. The Pearson correlation was used as the distance metric and average linkage clustering as the linkage method.

### 2.9. cDNA Synthesis and RT-PCR

cDNA was synthesised from the total RNA (see the “Total RNA Isolation” section) using PrimeScript™ RT (Takara Bio Inc., Otsu, Japan), and the resulting cDNA was amplified using TB Green^®^ Premix Ex Taq™ II (Tli RNaseH Plus) (Takara Bio Inc., Otsu, Japan). RT-PCR was performed at 95 °C for 30 s, followed by 40 cycles alternating between 95 °C for 5 s and 60 °C for 30 s, using the Thermal Cycler Dice^®^ Real-Time System (Takara Bio Inc., Otsu, Japan). mRNA levels were calculated using the cycle time (*Ct*) values normalised to β-actin (Actβ) values. Using the 2^–ΔΔCt^ method [26], samples were compared using relative quantification (fold change) values.

### 2.10. Statistical Analysis

R statistical software (v3.4.2) was used for the analyses. All data are presented as the mean ± standard error. Between-group differences were assessed using non-parametric Wilcoxon tests, and results with *p* < 0.05 were considered significant. To detect significantly over-represented GO categories and to characterise functionally regulated gene groups, we used Fisher’s exact test.

## 3. Results

### 3.1. Pharmacokinetics of High-Dose CY in Mice

CY and metabolite levels were measured in plasma samples of *C57BL/6J* mice that were administered 400–700 mg/kg CY. Figure S1 shows the concentration–time profiles for CY, HCY, and CEPM, as well as the mice survival estimates. At 1 h after the administration of CY (400 mg/kg), the blood CY level was 25.3 μg/mL, whereas 4 h after administration, it was <1.50 μg/mL (below the limit of measurement). The corresponding levels of HCY and CEPM were 7.4 and 0.6 μg/mL, and 26.5 and 4.0 μg/mL, respectively. The AUC values for CY, HCY, and CEPM were 246.4, 73.3, and 269.1 μM·h, respectively. Meanwhile, at 1 and 3 h after the administration of 500 mg/kg CY, the plasma levels of CY, HCY, and CEPM were 118.6 and 2.3, 92.4 and 3.0, and 69.1 and 16.9 μg/mL, respectively. The mean AUC values for CY, HCY, and CEPM were 1555.3, 1092.5, and 679.8 μM·h, respectively. Furthermore, for a dose of 700 mg/kg, the CY, HCY, and CEPM levels were 251.2 ± 94.1 and 5.2 ± 6.4 μM·h, 57.7 ± 10.6 and 5.9 ± 4.4 μM·h, and 67.2 ± 40.0 and 26.4 ± 17.4 μM·h, respectively. The mean AUC values for CY, HCY, and CEPM were 4809.2 ± 1782.0, 555.3 ± 115.1, and 1305.4 ± 254.9 μM·h, respectively. In *C57BL/6J* mice administered 400, 500, or 700 mg/kg CY intraperitoneally, HCY and CEPM were produced in a manner comparable to that observed in humans (Figure S2). Various doses of CY were administered to the mice, and the maximum dose of CY that allowed the mice to survive for more than a week was determined to be 400 mg/kg/day for two days, which was a part of the administration schedule in this study (Figure S1).

### 3.2. Histopathological Examination 

**Figure 3:**
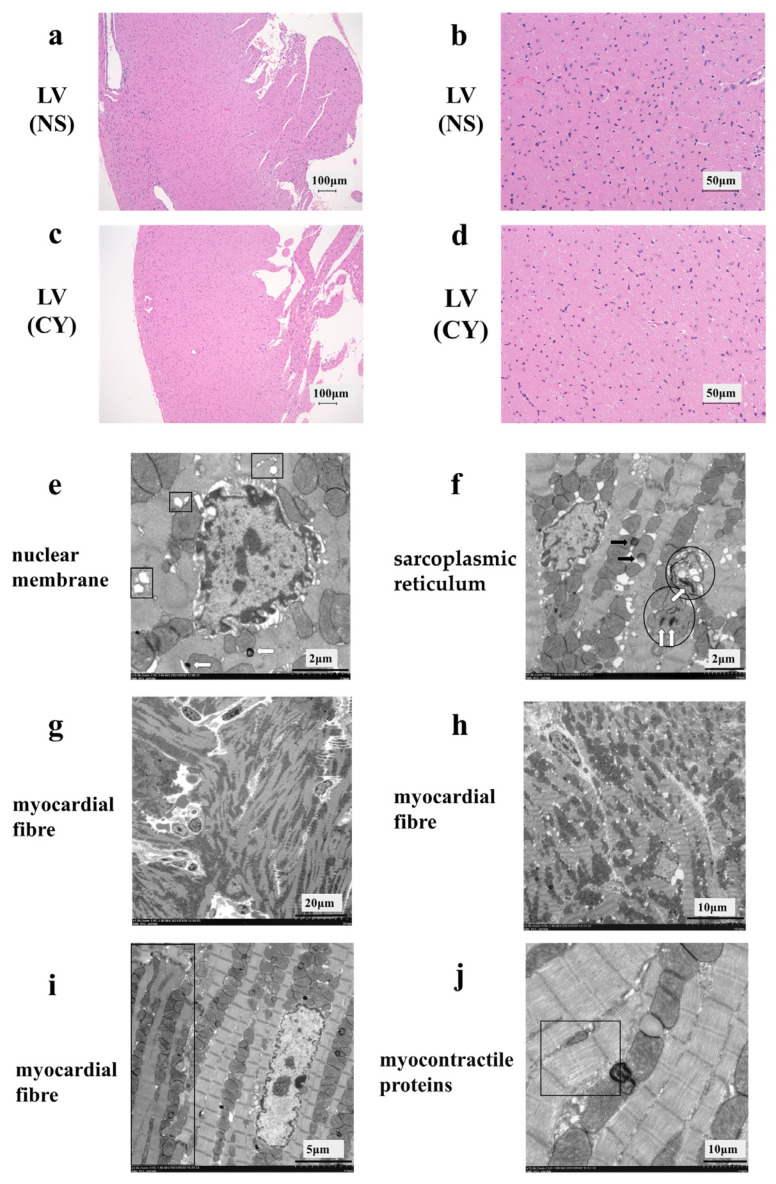
Effect of cyclophosphamide (CY) on mouse cardiac tissue. Light microscopic examination of the haematoxylin- and eosin-stained left ventricle cardiac tissue of mice treated with saline ((**a**,**b**), 100× magnification) and mice treated with CY ((**c**,**d**), 400× magnification). Images (**b**,**d**) are magnified versions of images (**a**,**c**), respectively. Panels (**e**–**j**) show images of the ultra-structure of the cardiac tissue of the mice treated with CY. The squares in panel (**e**) show the expansion of transverse tubules and the sarcoplasmic reticulum. Arrows defined by outlines in panels (**e**,**f**) show lamellar bodies. The circled areas in (**f**) show mitochondria that have either lost or expanded their internal structure. The black arrows in panel (**f**) show lipid droplets. Panels (**f,g**) show the turbulence of myocardial fibre travel. The dark area surrounded by a square shows muscle contractile fibre structures with different contractile states from within the same muscle cell. Panel (**j**) shows low-density contractile proteins in a cardiomyocyte. LV: left ventricle, NS: normal saline.

HE staining showed no difference between the CY-treated and NS-treated groups (Figure 3a–d). However, the electron micrograph analysis revealed ultrastructural alterations in the cardiac tissue of mice treated with CY. Treatment with CY (400 mg/kg × 2 days) caused marked ultrastructural aberrations (Figure 3e–j). The nuclear membrane cavity showed generalised dissociation and localised heavy dilation (Figure 3e). Lamellar bodies appeared in the cytoplasm, and the expansion of the transverse tubule and sarcoplasmic reticulum was observed (Figure 3e,f). Mitochondrial damage was observed, including an expansion in size, the disintegration of cristae, and the loss of the matrix (Figure 3f). Fat droplets and vacuolar degeneration were also observed (Figure 3f). In addition, lamellar bodies were found in the damaged mitochondria (Figure 3f). Turbulence in the myocardial fibre travel was observed (Figure 3g,h). Furthermore, in the same myocardial cells, muscle contractile fibre structures were present, with contractile states different from those observed in other parts (Figure 3i). Low-density contractile proteins were detected in cardiomyocytes (Figure 3j). We measured the diameter and area of 100 mitochondria from electron microscope images taken at 7000 times magnification in each group using ImageJ software 1.53 (National Institutes of Health) (https://imagej.net/ij/, accessed on 20 April 2024). The area of the mitochondria in the CY-treated group (median 118.5 ± 1.5 μm^2^) was significantly smaller than that in the control group (median 128.5 ± 1.1 μm^2^) (*p* value < 0.01). However, no significant difference was observed between the diameters of the mitochondria in the CY-treated group (median 1.17 ± 0.04 μm) and the control group (median 1.18 ± 0.03 μm) (*p* value = 0.87).

### 3.3. Identification of Gene Expression Profiles after the Administration of High-Dose CY 

**Figure 4:**
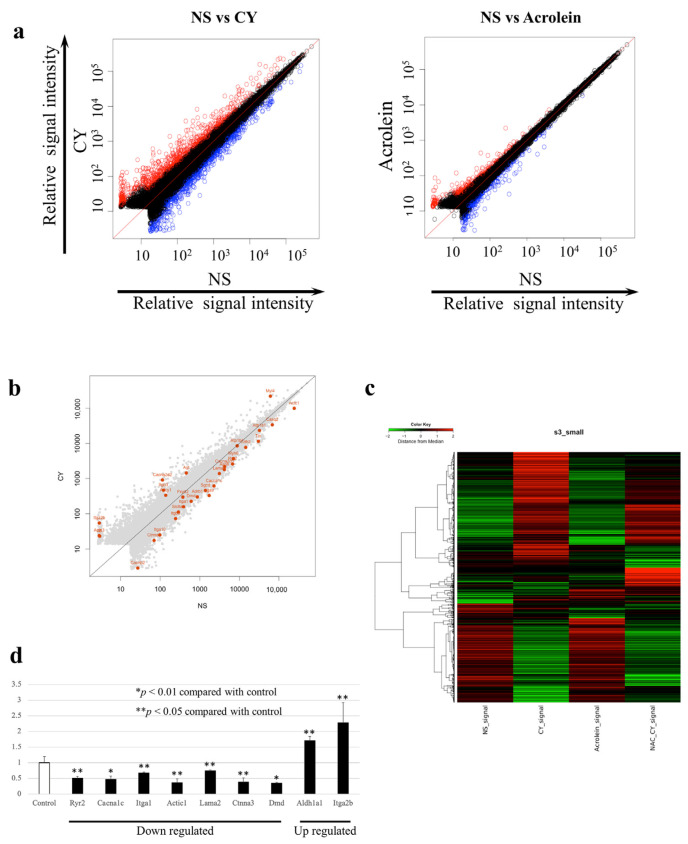
Gene expression profiles. (**a**) Scatterplots of gene expression in *C57BL/6J* mice administered cyclophosphamide (CY) or acrolein. The X-axis shows the relative normalised log_2_-signal intensity of the control (NS) samples and the Y-axis shows the normalised log_2_-signal intensity of the CY- or acrolein-treated samples. (**a**) Blue dots and red dots indicate downregulated and upregulated genes, respectively. (**b**) Scatterplots highlighting genes associated with myocardial contraction. (**c**) Clustering diagram of gene trees and a heatmap generated using MeV software 4.9.0 with the hierarchical clustering (HCL) method to sort the genes, with Pearson correlation estimates used as the distance metrics and average linkage clustering used as the linkage method. Red and green blocks represent high and low levels of expression, respectively, relative to those in the control group; black blocks indicate expression levels similar to those in the control. (**d**) Expression levels of genes associated with myocardial contraction after CY administration, as determined using RT-PCR.

We examined changes in the expression of 52,141 genes using whole-gene microarray analysis. The data were deposited at the National Center for Biotechnology Information Gene Expression Omnibus [27] and are accessible through the Gene Expression Omnibus Series (Accession Number GSE194073). At seven days after CY administration, the expression of 675 genes was significantly upregulated, whereas the expression of 535 genes was significantly downregulated compared with the control values. Table 1 shows the 20 most upregulated and 20 most downregulated genes after CY administration. At seven days after the administration of acrolein, 280 genes were significantly upregulated and 299 genes were significantly downregulated. Between-group differences in gene expression were examined using scatterplot and clustering analyses (Figure 4a,b). The number of genes with altered expression in the CY-treated mice was higher than that in the acrolein-treated mice (Figure 4a). Figure 4b presents the genes involved in myocardial contraction in mouse cardiac tissue expressed at 3 h after the administration of CY. Figure 4c shows the gene expression patterns observed in samples treated with CY, including heatmaps of the genes that differed in expression between the CY and NS groups. Furthermore, the pattern of the heatmap observed after CY administration was partially altered by the NAC treatment. The pattern of the heatmap observed after acrolein administration was similar to that after NS administration. 

### 3.4. Gene-Enrichment and Functional Annotation Analyses

The initial data were generated based on the results of the NS- and CY-administration groups and were categorised based on several annotation categories, including protein–protein interactions and GO terms (*p* < 0.05). The five most frequent functional annotation clustering categories obtained for the samples treated with CY for 3 h are shown in Table 2. Genes related to dilated cardiomyopathy, arrhythmogenic right ventricular cardiomyopathy, and hypertrophic cardiomyopathy, which is associated with myocardial dysfunction, were significantly altered by the administration of CY. The generation of functionally regulated gene groups was based on the KEGG pathway database (Table 3). Three hours after the administration of CY, the major genes identified in *C57BL/6J* mouse cardiac tissue were those involved in dilated cardiomyopathy, hypertrophic cardiomyopathy, arrhythmogenic right ventricular cardiomyopathy, viral protein interactions with cytokines and their receptors, and extracellular matrix receptor interactions.

### 3.5. RT-PCR Validation of the Microarray Analysis Findings

**Figure 5:**
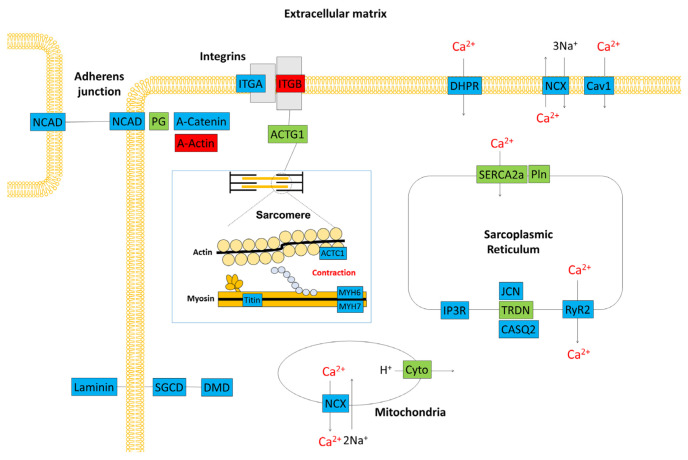
Schematic representation of the gene expression profile in the cardiac tissue of a *C57BL/6J* mouse. Red boxes denote genes that were upregulated 3 h after treatment with cyclophosphamide. Blue boxes denote downregulated genes. Green boxes show genes with unchanged expression. ACTC1: actin, alpha cardiac muscle, ACTG1: actin beta/gamma 1, CASQ2: calsequestrin 2, Cav1: voltage-dependent calcium channel L type alpha-1C, Cyto: ubiquinol–cytochrome c reductase iron–sulfur subunit, DHPR: voltage-dependent calcium channel L type alpha-1C, DMD: dystrophin, IP3R: inositol 1,4,5-triphosphate receptor type 1, ITGA: integrin alpha 1, ITGB: integrin beta 1, JCN: aspartate beta-hydroxylase, MYH6: myosin heavy chain 6, MYH7: myosin heavy chain 7, NCAD: cadherin 2, type 1, N-cadherin, NCX: solute carrier family 8 (sodium/calcium exchanger), PG: junction plakoglobin, Pln: phospholamban, RyR2: ryanodine receptor 2, SERCA2a: P-type Ca^2+^ transporter type 2A, SGCD: delta-sarcoglycan, TRDN: triadin.

Using RT-PCR and β-actin as a control, we examined the expression levels of nine genes, including those that are important for myocardial function. The RT-PCR data confirmed the results of the microarray analysis (Figure 4d). Figure 5 is a schematic diagram showing genes that are important for myocardial contraction in cardiomyocytes, with colour-coded changes in expression.

## 4. Discussion

We examined the ultrastructural alterations induced by the administration of high-dose CY in cardiac tissues using electron microscopic analysis. We found several structural changes in the cardiomyocyte nuclear membrane, sarcoplasmic reticulum, mitochondria, and muscle fibres after high-dose CY administration. Changes in the expression of genes associated with high-dose CY administration were detected via a comprehensive gene expression microarray analysis. Notably, most gene groups related to myocardial contraction and the Ca^2+^ signalling pathways were downregulated. The changes observed in these gene groups were consistent with those observed in the electron microscopy analysis. Genes that were altered by the administration of CY included genes related to oxidative stress, endoplasmic reticulum stress, apoptosis, *p53* expression, *p38MAPK*, GSK-3β, and Akt/PI3K signalling. However, there have been no reports on the myocardial contraction or Ca^2+^ signalling pathway [28,29,30,31,32,33] that may be involved in the mechanism of CY myocardial damage.

The pumping action of the heart involves the contraction and relaxation of cardiomyocytes, which constitute the heart. These mechanisms are mainly controlled by increases and decreases in intracellular Ca^2+^ concentrations. The regulation of intracellular Ca^2+^ concentrations is important for the heart to continuously and smoothly pump blood throughout the body [34,35]. The increase in intracellular Ca^2+^ concentrations in the myocardium is triggered by an influx of Ca^2+^ through the L-type Ca^2+^ channels (DHPR) and Ca^2+^ release from the sarcoplasmic reticulum (RyR2) upon stimulation [36,37]. The excess intracellular Ca^2+^ binds to troponin C (TnC), a subunit of the contractile regulatory protein troponin, and changes the steric configuration of tropomyosin, resulting in an interaction between actin and myosin. A contraction is induced by the interaction between actin and myosin. The decrease in the intracellular Ca^2+^ concentration is caused by the uptake of sarco/endoplasmic reticulum Ca^2+^-ATPase 2A into the sarcoplasmic reticulum, the extracellular efflux of the Na^+^/Ca^2+^ exchange system, or efflux by cell membrane Ca^2+^ pumps, resulting in the relaxation of the myocardium [38,39]. In our study, the gene expression of DHPR, RyR2, and TnC, which are important proteins involved in the regulation of the Ca^2+^ concentration, was decreased. The disruption of calcium regulatory mechanisms may cause elevated intracellular calcium concentrations, resulting in inflammation and the apoptosis of cardiomyocytes, leading to myocardial contractile dysfunction. Based on the new mechanism identified in this study, CY myocardial injury may be prevented with the use of Ca^2+^ signalling sensitisers, such as levosimendan and pimobendan [40].

Concurrently, the gene expression analysis conducted after the administration of acrolein, which is considered to be the main driver of CY-related myocardial damage in in vitro research, revealed patterns that were different from those observed after the administration of CY. Acrolein is a small molecule that is highly reactive when exposed to unsaturated aldehyde and is quickly adsorbed by proteins and other substances [16,17]. Therefore, it may be necessary to re-examine whether the acrolein dosage and method of administration used in this study were appropriate. Furthermore, given the metabolism of CY, the myocardial injury model of CY may not be exactly replicated unless acrolein is present in the myocardial cells. The findings of the present study preclude us from making any conclusions regarding the contribution of acrolein to CY cardiotoxicity.

The NAC treatment significantly altered the expression of genes related to pathways such as chemical cardiogenesis–DNA adducts, complement and coagulation cascades, and retinol metabolism. The NAC treatment also significantly altered the expression of genes related to dilated cardiomyopathy, hypertrophic cardiomyopathy, and arrhythmogenic right ventricular cardiomyopathy. The findings of the electron microscopy analysis conducted after the administration of NAC were comparable to those achieved after CY administration. The experimental methods used to evaluate the NAC treatment require further research.

ST2, a member of the interleukin-1 receptor family [41], is released by cardiomyocytes in response to myocardial stress [42]. ST2 levels were reported to be increased and were associated with changes in left ventricular systolic function during a three-year follow-up period after adjuvant radiotherapy for breast cancer [43]. ST2 levels are a potential new biomarker in CY-induced cardiotoxicity.

Our study had several limitations. First, all of the mice used in this study were young females. This is because cyclophosphamide-induced cardiotoxicity is often a clinical problem in young females. However, more animal models are worthy of study. Second, it is unclear whether the highly reactive acrolein properly affected the cardiomyocytes of mice in the present exposure procedure.

## 5. Conclusions

At high doses, CY causes the expansion of transverse tubules and the sarcoplasmic reticulum, leads to turbulence in myocardial fibre travel, and generates low-density contractile proteins in cardiomyocytes, as observed using electron microscopy. The microarray analysis revealed that high-dose CY treatment changed the cardiomyocyte expression of 1210 genes associated with cardiomyopathy and cardiac muscle function. The results of the gene expression profiles, along with functional annotation clustering and the KEGG pathway functional classification analysis, suggest that CY-induced cardiotoxicity is associated with the disruption of the Ca^2+^ signalling pathway; these findings are consistent with the results of the electron microscopy. The CY-induced cardiotoxicity identified in this study may help to prevent adverse cardiac events during treatment by avoiding the disruption of Ca^2+^ regulatory mechanisms.

## Figures and Tables

**Table 1:** Previous reports of HL *C57BL/6J* mice treated with CY: the 20 most up- and downregulated genes among the 52,142 genes examined.

**Upregulated genes:**			
**Gene Symbol**	**Acc#**	**Z score**	**Gene name**
*Hmox1*	NM_010442	9.075	Haem oxygenase 1
*Hamp*	NM_032541	8.900	Hepcidin antimicrobial peptide
*Mt2*	NM_008630	8.579	Metallothionein 2
*Serpina3n*	NM_009252	8.301	Serine (or cysteine) peptidase inhibitor clade A, member 3N
*Dmkn*	NM_001166173	7.662	Dermokine, transcript variant 3
*Cd207*	NM_144943	7.595	CD207 antigen
*Dkk3*	NM_015814	7.498	Dickkopf WNT signalling pathway inhibitor 3, transcript variant 2
*Mlana*	XM_011247400	7.345	Melan-A
*Sfrp5*	NM_018780	7.028	Secreted frizzled-related sequence protein 5
*Pip5k1a*	AK167816	6.856	Phosphatidylinositol-4-phosphate 5-kinase, type 1 alpha
*Clec1b*	NM_019985	6.784	C-type lectin domain family 1, member b, transcript variant 1
*Anxa8*	NM_013473	6.760	Annexin A8, transcript variant 1
*Fam32a*	NM_026455	6.494	Family with sequence similarity 32, member A
*Gp9*	NM_018762	6.430	Glycoprotein 9 (platelet)
*Lgals3*	NM_001145953	6.298	Lectin, galactose binding, soluble 3, transcript variant 1
*Fam183b*	NM_029283	6.237	Family with sequence similarity 183, member B transcript variant 1
*Clec4d*	NM_010819	6.155	C-type lectin domain family 4, member d, transcript variant 1
*Cntf*	NM_170786	6.133	Ciliary neurotrophic factor
*Ppbp*	NM_023785	6.065	Pro-platelet basic protein
*Gata2*	NM_008182	5.986	Glutathione S-transferase, alpha 2
**Downregulated genes:**			
**Gene Symbol**	**Acc#**	**Z score**	**Gene name**
*Cx3cr1*	NM_009987	−6.630	Chemokine (C-X3-C motif) receptor 1
*Nckap5*	NM_172484	−5.346	NCK-associated protein 5, transcript variant 2
*Aplnr*	NM_011784	−5.227	Apelin receptor
*Apln*	NM_013912	−5.118	Apelin
*Itga9*	NM_133721	−4.791	Integrin alpha 9, transcript variant 1
*Tfrc*	NM_011638	−4.554	Transferrin receptor, transcript variant 1
*Ppargc1b*	NM_133249	−4.504	Peroxisome proliferative activated receptor, gamma, coactivator 1 beta, transcript variant 1
*Akap7*	XM_006512789	−4.497	A kinase (PRKA) anchor protein 7
*Cops8*	NM_133805	−4.479	COP9 signalosome subunit 8
*Plekhh1*	NM_181073	−4.462	Pleckstrin homology domain containing, family H (with MyTH4 domain) member 1
*Galnt17*	NM_145218	−4.418	Polypeptide N-acetylgalactosaminyltransferase 17
*Large1*	NM_010687	−4.360	LARGE xylosyl- and glucuronyltransferase 1, transcript variant 2
*Klhdc8a*	NM_144810	−4.358	Kelch domain containing 8A
*Kcne1*	NM_008424	−4.307	Potassium voltage-gated channel, lsk-related subfamily, member 1
*Nrn1*	NM_001374754	−4.275	Neuritin 1, transcript variant 2
*Klra8*	NM_010650	−4.228	Killer cell lectin-like receptor, subfamily *A, member 8, transcript variant 2*
*Ccl21a*	NM_011124	−4.226	Chemokine (C-C motif) ligand 21A (serine)
*Dbp*	NM_016974	−4.233	D site albumin promoter binding protein
*Fhl2*	NM_010212	−4.201	Four and a half LIM domains 2, transcript variant 1
*Tuba4a*	NM_009447	−4.169	Tubulin, alpha 4A, transcript variant 1

**Table 2:** Functional annotation clustering analysis of genes expressed in *C57BL/6J* mouse cardiac tissue 3 h after the last dose of CY.

Annotation Cluster	Count	*p*
1 (Enrichment Score: 20.72)		
Extracellular region	210	2.8 × 10^−29^
Extracellular space	196	1.3 × 10^−20^
Signal	349	7.0 × 10^−12^
2 (Enrichment Score: 13.13)		
Signal	349	7.0 × 10^−12^
Disulphide bond	249	2.3 × 10^−11^
Glycoprotein	345	4.9 × 10^−11^
3 (Enrichment Score: 7.52)		
Cell junction	89	2.3 × 10^−9^
Synapse	55	4.0 × 10^−7^
4 (Enrichment Score: 6.14)		
Dilated cardiomyopathy	21	3.1 × 10^−7^
Hypertrophic cardiomyopathy	20	8.5 × 10^−7^
Arrhythmogenic right ventricular cardiomyopathy	18	1.4 × 10^−6^
5 (Enrichment Score: 4.00)		
Membrane	451	1.9 × 10^−12^
Plasma membrane	361	1.2 × 10^−6^
Cell membrane	237	4.8 × 10^−5^

**Table 3:** KEGG pathway functional classification of genes in *C57BL/6J* mouse cardiac tissue expressed 3 h after the administration of CY.

Term	Count	*p*
Dilated cardiomyopathy	21	3.1 × 10^−7^
Hypertrophic cardiomyopathy (HCM)	20	8.5 × 10^−7^
Arrhythmogenic right ventricular		
Cardiomyopathy (ARVC)	18	1.4 × 10^−6^
Viral protein interaction with cytokine		
and cytokine receptor	20	1.7 × 10^−6^
ECM-receptor interaction	19	2.2 × 10^−6^
Focal adhesion	29	1.3 × 10^−5^
Metabolism of xenobiotics by cytochrome P450	16	1.5 × 10^−5^
Hematopoietic cell lineage	18	2.4 × 10^−5^
Fluid shear stress and atherosclerosis	23	4.1 × 10^−5^
Platinum drug resistance	16	4.6 × 10^−5^

## Data Availability

The datasets and plugins used for data processing in this study are available from T.N. (adu44150@ams.odn.ne.jp) on reasonable request.

## Supplementary Materials

The following supporting information can be downloaded at: https://www.mdpi.com/article/10.3390/diseases12050085/s1. Figure S1: Protocol for measuring blood levels of cyclophosphamide (CY) and its metabolites in *C57BL/6J* mice and the effect of CY administration; Figure S2: Pharmacokinetics of high-dose cyclophosphamide (CY) in *C57BL/6J* mice.

## Abbreviations

Abbreviation	Full term or phrase
Actβ	actin beta
Akt	protein kinase B
AldoCY	aldocyclophosphamide
ALDH1	aldehyde dehydrogenase 1
AUC	area under the concentration-time curve
CEPM	o-carboxyethyl-phosphoramide mustard
CY	cyclophosphamide
CYP	cytochrome P-450
Ct	cycle time
DAVID	Database for Annotation, Visualization, and Integrated Discovery
EDTA	ethylenediaminetetraacetic acid
GO	gene ontology
GSK-3β	glycogen synthase kinase 3β
HE	haematoxylin-Eosin
HCL	hierarchical clustering
4-HCY	4-hydroxy-cyclophosphamide
HSCT	hematopoietic stem cell transplantation
i.p.	Intraperitoneally
KEGG	Kyoto Encyclopedia of Genes and Genomes
LC/MS/MS	liquid chromatography/tandem mass spectrometry
NAC	N-acetylcysteine
NS	normal saline
p38MAPK	P38 mitogen-activated protein kinase
PI3K	Phosphoinositide 3-kinase
PM	phosphoramide mustard
RT-PCR	reverse transcription PCR
TnC	troponin C

## References

[1] Santos G.W., Sensenbrenner L.L., Burke P.J., Mullins G.M., Blas W.B., Tutschka P.J., Slavin R.E. (1972). The use of cyclophosphamide for clinical marrow transplantation. Transplant. Proc..

[2] Emadi A., Jones R.J., Brodsky R.A. (2009). Cyclophosphamide and cancer: Golden anniversary. Nat. Rev. Clin. Oncol..

[3] Vivarelli M., Gibson K., Sinha A., Boyer O. (2023). Childhood nephrotic syndrome. Lancet.

[4] O’Donnell P.V., Luznik L., Jones R.J., Vogelsang G.B., Leffell M.S., Phelps M., Rhubart P., Cowan K., Piantados S., Fuchs E.J. (2002). Nonmyeloablative bone marrow transplantation from partially HLA-mismatched related donors using posttransplantation cyclophosphamide. Biol. Blood Marrow Transplant..

[5] Nishikawa T. (2024). Human leukocyte antigen-haploidentical haematopoietic stem cell transplantation using post-transplant cyclophosphamide for paediatric haematological malignancies. Cancers.

[6] Atalay F., Gulmez O., Ozsancak Ugurlu A. (2014). Cardiotoxicity following cyclophosphamide therapy: A case report. J. Med. Case Rep..

[7] Dhesi S., Chu M.P., Blevins G., Paterson I., Larratt L., Oudit G.Y., Kim D.H. (2013). Cyclophosphamide-induced cardiomyopathy: A case report, review, and recommendations for management. J. Investig. Med. High Impact Case Rep..

[8] Krüger-Genge A., Köhler S., Laube M., Haileka V., Lemm S., Majchrzak K., Kammerer S., Schulz C., Storsberg J., Pietzsch J. (2023). Anti-Cancer Prodrug Cyclophosphamide Exerts Thrombogenic Effects on Human Venous Endothelial Cells Independent of CYP450 Activation-Relevance to Thrombosis. Cells.

[9] Gottdiener J.S., Appelbaum F.R., Ferrans V.J., Deisseroth A., Ziegler J. (1981). Cardiotoxicity associated with high-dose cyclophosphamide therapy. Arch. Intern. Med..

[10] McDonald G.B., Slattery J.T., Bouvier M.E., Ren S., Batchelder A.L., Kalhorn T.F., Schoch H.G., Anasetti C., Gooley T. (2003). Cyclophosphamide metabolism, liver toxicity, and mortality following hematopoietic stem cell transplantation. Blood.

[11] Nishikawa T., Miyahara E., Kurauchi K., Watanabe E., Ikawa K., Asaba K., Tanabe T., Okamoto Y., Kawano Y. (2015). Mechanisms of fatal cardiotoxicity following high-dose cyclophosphamide therapy and a method for its prevention. PLoS ONE.

[12] Kurauchi K., Nishikawa T., Miyahara E., Okamoto Y., Kawano Y. (2017). Role of metabolites of cyclophosphamide in cardiotoxicity. BMC Res. Notes.

[13] Yoshida M., Tomitori H., Machi Y., Hagihara M., Higashi K., Goda H., Ohya T., Niitsu M., Kashiwagi K., Igarashi K. (2009). Acrolein toxicity: Comparison with reactive oxygen species. Biochem. Biophys. Res. Commun..

[14] Lau S., Rangarajan R., Philidet C., Krüger-Genge A., Braune S., Kammerer S., Küpper J.H., Lendlein A., Jung F. (2020). Effects of acrolein in comparison to its prodrug cyclophosphamide on human primary endothelial cells in vitro. Toxicol. In Vitro.

[15] Dionísio F., Araújo A.M., Duarte-Araújo M., Bastos M.L., Guedes de Pinho P., Carvalho F., Costa V.M. (2022). Cardiotoxicity of cyclophosphamide’s metabolites: An in vitro metabolomics approach in AC16 human cardiomyocytes. Arch. Toxicol..

[16] Podgurskaya A.D., Slotvitsky M.M., Tsvelaya V.A., Frolova S.R., Romanova S.G., Balashov V.A., Agladze K.I. (2021). Cyclophosphamide arrhythmogenicitytesting using human-induced pluripotent stem cell-derived cardiomyocytes. Sci. Rep..

[17] Liu W., Zhai X., Wang W., Zheng B., Zhang Z., Fan X., Chen Y., Wang J. (2018). Aldehyde dehydrogenase 2 activation ameliorates cyclophosphamide-induced acute cardiotoxicity via detoxification of toxic aldehydes and suppression of cardiac cell death. J. Mol. Cell. Cardiol..

[18] Muguruma K., Pradipta A.R., Ode Y., Terashima K., Michiba H., Fujii M., Tanaka K. (2020). Disease-associated acrolein: A possible diagnostic and therapeutic substrate for in vivo synthetic chemistry. Bioorg. Med. Chem..

[19] Kehrer J.P., Biswal S.S. (2000). The molecular effects of acrolein. Toxicol. Sci..

[20] Bolstad B.M., Irizarry R.A., Astrand M., Speed T.P. (2003). A comparison of normalization methods for high density oligonucleotide array data based on variance and bias. Bioinformatics.

[21] Gentleman R.C., Carey V.J., Bates D.M., Bolstad B., Dettling M., Dudoit S., Ellis B., Gautier L., Ge Y., Gentry J. (2004). Bioconductor: Open software development for computational biology and bioinformatics. Genome Biol..

[22] Quackenbush J. (2002). Microarray data normalization and transformation. Nat. Genet..

[23] Huang D.W., Sherman B.T., Lempicki R.A. (2009). Systematic and integrative analysis of large gene lists using DAVID bioinformatics resources. Nat. Protoc..

[24] Kanehisa M., Goto S. (2000). KEGG: Kyoto Encyclopedia of Genes And Genomes. Nucleic Acids Res..

[25] Saeed A.I., Sharov V., White J., Li J., Liang W., Bhagabati N., Braisted J., Klapa M., Currier T., Thiagarajan M. (2003). TM4: A free, open-source system for microarray data management and analysis. BioTechniques.

[26] Livak K.J., Schmittgen T.D. (2001). Analysis of relative gene expression data using real-time quantitative PCR and the 2^−ΔΔCt^ method. Methods.

[27] Edgar R., Domrachev M., Lash A.E. (2002). Gene expression omnibus: NCBI gene expression and hybridization array data repository. Nucleic Acids Res..

[28] Iqubal A., Iqubal M.K., Sharma S., Ansari M.A., Najmi A.K., Ali S.M., Ali J., Haque S.E. (2019). Molecular mechanism involved in cyclophosphamide-induced cardiotoxicity: Old drug with a new vision. Life Sci..

[29] Song Y., Zhang C., Wang C., Zhao L., Wang Z., Dai Z., Lin S., Kang H., Ma X. (2016). Ferulic acid against cyclophosphamide-induced heart toxicity in mice by inhibiting NF-κB pathway. Evid. Based Complement. Altern. Med..

[30] Fatani A.G., Darweesh A.Q., Rizwan L., Aleisa A.M., Al-Shabanah O.A., Sayed-Ahmed M.M. (2010). Carnitine deficiency aggravates cyclophosphamide-induced cardiotoxicity in rats. Chemotherapy.

[31] Asiri Y.A. (2010). Probucol attenuates cyclophosphamide-induced oxidative apoptosis, p53 and Bax signal expression in rat cardiac tissues. Oxid. Med. Cell. Longev..

[32] Park E.S., Kang J.C., Jang Y.C., Park J.S., Jang S.Y., Kim D.E., Kim B., Shin H.S. (2014). Cardioprotective effects of rhamnetin in H9c2 cardiomyoblast cells under H_2_O_2_-induced apoptosis. J. Ethnopharmacol..

[33] El-Agamy D.S., Elkablawy M.A., Abo-Haded H.M. (2017). Modulation of cyclophosphamide-induced cardiotoxicity by methyl palmitate. Cancer Chemother. Pharmacol..

[34] Bers D.M. (2001). Excitation-Contraction Coupling and Cardiac Contractile Force.

[35] Avila G., de la Rosa J.A., Monsalvo-Villegas A., Montiel-Jaen M.G. (2019). Ca^2+^ channels mediate bidirectional signaling between sarcolemma and sarcoplasmic reticulum in muscle cells. Cells.

[36] Protasi F. (2002). Structural interaction between RYRs and DHPRs in calcium release units of cardiac and skeletal muscle cells. Front. Biosci..

[37] Ríos E., Figueroa L., Manno C., Kraeva N., Riazi S. (2015). The couplonopathies: A comparative approach to a class of diseases of skeletal and cardiac muscle. J. Gen. Physiol..

[38] Rossi A.E., Dirksen R.T. (2006). Sarcoplasmic reticulum: The dynamic calcium governor of muscle. Muscle Nerve.

[39] Tadini-Buoninsegni F., Smeazzetto S., Gualdani R., Moncelli M.R. (2018). Drug interactions with the Ca^2+^-ATPase from sarco (Endo) plasmic reticulum (SERCA). Front. Mol. Biosci..

[40] Endoh M. (2008). Cardiac Ca^2+^ signaling and Ca^2+^ sensitizers. Circ. J..

[41] Pascual-Figal D.A., Januzzi J.L. (2015). The Biology of ST2: The International ST2 Consensus Panel. Am. J. Cardiol..

[42] Riccardi M., Myhre P.L., Zelniker T.A., Metra M., Januzzi J.L., Inciardi R.M. (2023). Soluble ST2 in Heart Failure: A Clinical Role beyond B-Type Natriuretic Peptide. J. Cardiovasc. Dev. Dis..

[43] Aula H., Skyttä T., Tuohinen S., Luukkaala T., Hämäläinen M., Virtanen V., Raatikainen P., Moilanen E., Kellokumpu-Lehtinen P.L. (2020). ST2 levels increased and were associated with changes in left ventricular systolic function during a three-year follow-up after adjuvant radiotherapy for breast cancer. Breast.

