# Supplementary material for: Effects of High-Dose Cyclophosphamide on Ultrastructural Changes and Gene Expression Profiles in the Cardiomyocytes of C57BL/6J Mice
Source: Diseases. 2024 Apr 27;12(5):85. doi: 10.3390/diseases12050085 (PMC11120609; doi:10.3390/diseases12050085)
Supplement: Supplementary file 1 [file diseases-12-00085-s001.zip › diseases-2970989-supplementary.pdf]

Supplementary information

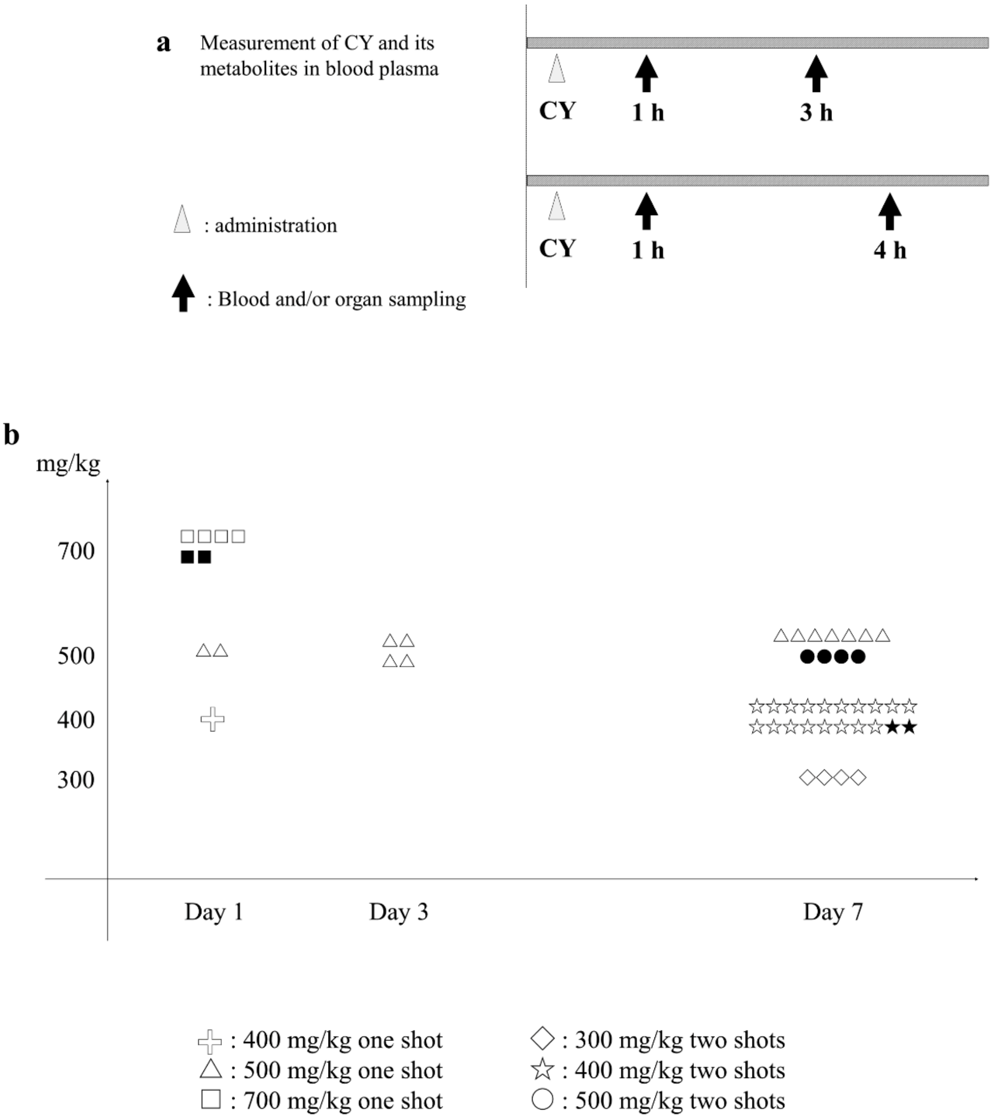

Figure S1. Protocol for measuring blood levels of cyclophosphamide (CY) and its metabolites in *C57BL/6J* mice and the effect of CY administration. (a) *C57BL/6J* mice (females aged 6 weeks) were intraperitoneally administered normal saline (control) or 400, 500, or 700 mg/kg CY once daily. One and three (4 h for the CY 400 mg/kg group) hours after normal saline or CY administration, blood samples were

collected from the vena caudalis. (b) Survival duration and surviving mouse count were recorded after CY administration. Black indicates dead mouse count until the last day of observation. White indicates surviving mice on the last day of observation.

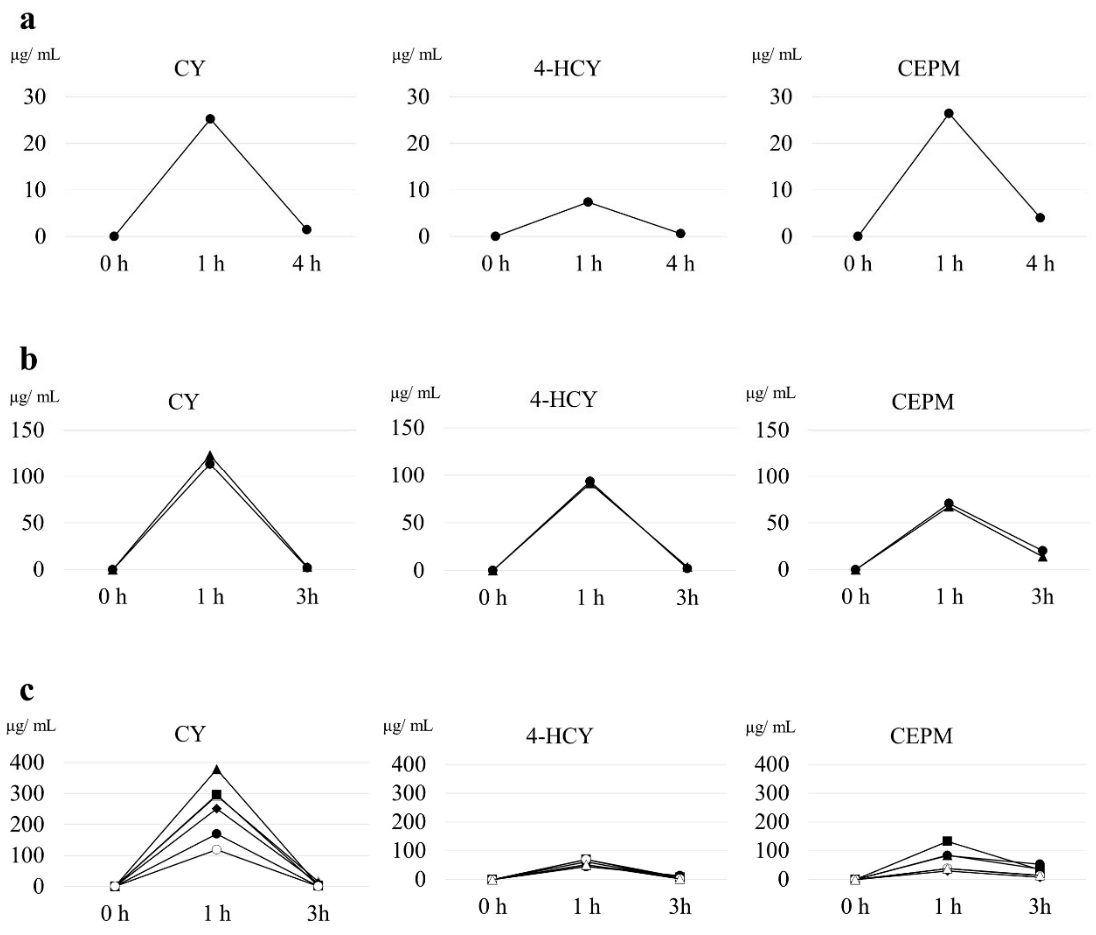

Figure S2. Pharmacokinetics of high-dose cyclophosphamide (CY) in *C57BL/6J* mice. (a) Time-dependent changes in blood levels of CY, 4-HCY, and CEPM after the administration of 400 mg/kg CY (n = 1). (b) Results of the group administered CY at a dose of 500 mg/kg (n = 2). (c) Results of the group administered CY at a dose of 700 mg/kg (n = 6).
